# Application of AI in Hypertension Health Education: Scoping Review

**DOI:** 10.2196/95596

**Published:** 2026-07-15

**Authors:** Haoran Chen, Shenglan Xiao, Tong Wan, Gui Li, Yanhong Peng, Zhimin Wang

**Affiliations:** 1 School of Nursing and The Second Affiliated Hospital, Hengyang Medical School, University of South China, 28 West Changsheng Road, Hengyang, China, 86 13974733239

**Keywords:** artificial intelligence, hypertension, health education, large language models, knowledge graph

## Abstract

**Background:**

Hypertension is a major global health challenge, and effective health education is crucial for improving patients’ self-management. Traditional health education approaches are often limited by insufficient personalization, accessibility, and scalability. Artificial intelligence (AI), including natural language processing, machine learning, and large language models (LLMs), offers promising solutions to address these limitations. However, evidence regarding AI applications in hypertension health education has not been comprehensively synthesized.

**Objective:**

This scoping review aimed to summarize the current evidence on AI applications in hypertension health education, and identify research gaps to inform future research and practice.

**Methods:**

This review followed the Joanna Briggs Institute methodology and PRISMA-ScR (Preferred Reporting Items for Systematic Reviews and Meta-Analyses Extension for Scoping Reviews) guidelines. Six databases (PubMed, Embase, Web of Science, Cochrane Library, CINAHL, and Scopus) were searched from January 2015 to June 2026. Eligibility criteria were developed using the participant-concept-context framework. Two reviewers independently conducted study screening and data extraction. Study designs were classified using the Mixed Methods Appraisal Tool framework. Consistent with scoping review methodology, no formal quality assessment was performed. Findings were synthesized narratively and presented using evidence gap maps, tables, and figures.

**Results:**

A total of 24 studies from 11 countries were included, comprising 6 randomized controlled trials, 4 nonrandomized trials, 11 quantitative descriptive studies, and 3 mixed methods studies. Most studies were published between 2024 and 2026. In total, 3 AI application scenarios were identified: rule-based health education, data-driven adaptive health education, and generative AI–driven health education. Natural language processing was the most widely applied technology, and LLM-based applications increased rapidly after 2023. However, generative AI studies were predominantly proof-of-concept evaluations and lacked randomized clinical validation. Health education was rarely implemented as a standalone intervention and was typically embedded within multifunctional AI platforms. Outcomes were categorized using the Digital Health Scorecard Framework across 4 domains: technology, clinical, usability, and cost. Technical accuracy and blood pressure outcomes were the most frequently reported measures, whereas no study evaluated economic outcomes.

**Conclusions:**

This first scoping review of AI applications in hypertension health education identified a mismatch between rapid advances in generative AI and the limited availability of rigorous clinical evidence. Three major research gaps were identified: (1) the lack of standardized core outcome sets covering technical, behavioral, clinical, and implementation domains; (2) limited development of hybrid architectures integrating LLM with structured medical knowledge bases; and (3) the absence of evaluation frameworks that satisfy both regulatory and implementation requirements. AI appears most suitable as a complement to, rather than a replacement for, clinician-delivered education. Future research should prioritize rigorous clinical validation, economic evaluation, multicultural adaptation, and health literacy equity to ensure that AI-driven health education reduces rather than exacerbates disparities in hypertension control.

## Introduction

### Rationale

Hypertension is a predominant global chronic condition and has become an important risk factor for many diseases [1]. The high and increasing global burden of hypertension presents a major health challenge, as it contributes to morbidity and mortality from cardiovascular and kidney diseases and imposes substantial financial costs on society [2]. Without effective interventions, the prevalence and absolute burden of hypertension will continue to rise [3,4].

The management of hypertension is a major challenge worldwide, with its control influenced by a variety of factors, including poor treatment adherence, inappropriate medication regimens, lifestyle, and socioeconomic status [5]. Some of these factors, such as poor adherence and unhealthy lifestyles, highlight a key issue: patients’ inadequate self-management skills [6]. Targeted health education is key to improving self-management skills and benefits patients [7,8]. In addition, numerous studies indicate that health education can be used as a tool to promote adherence to improve blood pressure control in patients with hypertension [9-13]. Health education is a continuous, dynamic, and planned teaching-learning process that spans the entire life cycle. Through an equal partnership between professionals and clients, it empowers individuals to proactively change their lifestyles in order to achieve positive health outcomes [14].

However, limited clinical staff and time resources often make it difficult to implement this ideal model on a large scale in practice, resulting in most health education remaining at the level of traditional, one-way, and brief information dissemination [15,16]. Traditional health education methods, such as brochures and verbal instruction, have several limitations: they are time-consuming, offer standardized content that lacks personalization, and make continuous follow-up difficult [17,18]. Therefore, new strategies and tools are needed, and recent advances in artificial intelligence (AI) present a promising solution to these limitations.

In recent years, AI has been applied across multiple fields of medicine, playing a crucial role in areas such as clinical decision support, medical image analysis, and genomics research [19]. With the advancement of technology, AI has also provided new approaches for the management of hypertension [4]. AI, particularly natural language processing (NLP), machine learning (ML), and large language model (LLM), holds immense potential for understanding patient needs, generating personalized content, and providing dynamic feedback [20,21]. Applying AI to hypertension health education could yield positive outcomes. Although AI has been widely applied in the field of hypertension, there remains a gap in the literature regarding how AI can enhance hypertension health education.

Several reviews have explored the role of AI in hypertension management and patient education. Aydin et al [22] conducted a scoping review on the application of LLM in patient education within the medical field. A 2025 review further explored the application of LLM in chronic disease management tasks, with patient education accounting for 62% of the included studies [23]. However, it did not specifically focus on hypertension. A recent scoping review in 2026 synthesized 33 studies on the application of LLM in hypertension care, emphasizing model optimization strategies and evaluation methods rather than AI as a health education tool [24]. Overall, while these reviews have explored the clinical utility, technical performance, or general applicability of AI in patient education, none have systematically examined the specific applications of AI technology in hypertension health education. This research gap urgently requires dedicated study.

### Objectives

For the reasons outlined earlier, systematically mapping the existing evidence on the use of AI in hypertension health education is warranted. Therefore, this scoping review aims to synthesize the published literature on the application of AI in hypertension health education. The specific objectives are (1) to identify the types of AI technologies used and their application scenarios, (2) to characterize the methodological approaches or research designs adopted in this domain, and (3) to catalog the outcome measures used to evaluate AI-based hypertension health education.

## Methods

### Overview

In this study, we used the Joanna Briggs Institute’s scoping review framework to map the research landscape regarding the application of AI technologies in health education for hypertension [25,26]. To ensure the reliability of our findings and their practical applicability, we conducted a comprehensive search, systematic screening, and structured data extraction. As this is a scoping review, no formal critical appraisal of study quality was performed [27]. This review is reported in accordance with the PRISMA-ScR (Preferred Reporting Items for Systematic Reviews and Meta-Analyses Extension for Scoping Reviews) guidelines [28], and a complete checklist is provided as Checklist 1.

### Protocol and Registration

The protocol for this scoping review was registered with the Open Science Framework (OSF registration number: 4wv3f).

### Eligibility Criteria

The inclusion criteria for this scoping review were based on the Joanna Briggs Institute Scope Review Methodology Guide and structured using the participants, concept, context framework. The inclusion and exclusion criteria are presented in Textbox 1.

Textbox 1. **Inclusion and exclusion criteria.**

**Inclusion criteria**

- Population: Adults (≥18 years) across the full hypertensive disease trajectory, from individuals with high-normal blood pressure at risk of developing hypertension to those with diagnosed and treated hypertension.
- Concept: Studies that used any form of artificial intelligence (AI) technology, defined as the use of computational methods to perform tasks that normally require human intelligence. Eligible AI technologies included, but were not limited to, machine learning, deep learning, natural language processing, large language models, expert systems, knowledge bases, and conversational agents.
- Context: Hypertension health education, defined as the provision of information, knowledge, or skills training, aimed at improving self-management, treatment adherence, medication adherence, lifestyle modification, or disease awareness among individuals across the hypertension spectrum. This encompasses knowledge dissemination, lifestyle guidance, health education material generation, and patient education delivered in any setting.
- Types of evidence sources: Peer-reviewed original research papers with full text available. No restrictions were placed on publication language.
- Study design: Randomized or nonrandomized controlled trials, qualitative and quantitative studies, and mixed methods studies.

**Exclusion criteria**

- Population: Studies that did not include participants within the hypertension disease spectrum, or studies that did not report relevant findings.
- Concept: Studies in which the intervention or technology did not involve an AI component as defined. For example, traditional web-based educational platforms without AI-driven functionality, standard telemonitoring without intelligent processing, or purely human-delivered education.
- Context: Studies addressing contexts other than hypertension health education or patient education for populations with hypertension. For example, AI applications exclusively for hypertension diagnosis, risk prediction, drug discovery, or clinical decision support without an educational component.
- Study types other than original research: Review, meta-analysis, editorial, commentary, letter, conference abstract, dissertation, book, book chapter, preprint, and protocol.
- Types of evidence sources: Study not subjected to peer review.

### Information Sources

We conducted a systematic search of the following 6 electronic databases: PubMed, Embase, Web of Science, Cochrane Library, CINAHL, and Scopus.

### Search

The search strategy for this study followed the PRISMA-S guidelines (an extension of the PRISMA [Preferred Reporting Items for Systematic Reviews and Meta-Analyses] statement for reporting literature searches in systematic reviews) [29]. The initial search was conducted in January 2026. Following iterative optimization of the search strategy, the Scopus database was added in May 2026, and a new search was performed. The search strategy was updated again in June 2026, and a second search was conducted to include the latest literature and any studies missed by the previous search strategy. The initial search strategy covered 5 databases: PubMed, Embase, Web of Science, Cochrane Library, and CINAHL. Detailed search strategies for all databases are provided in Multimedia Appendix 1. In accordance with the guidelines, this study used a 3-step search strategy. The first step involved a preliminary, limited-scope search of PubMed to analyze keywords in the titles and abstracts of relevant papers, as well as the index terms used to describe the papers, thereby developing a comprehensive search strategy. The second step involved a comprehensive search of all 6 databases using all identified keywords and index terms. To ensure reproducibility, the complete electronic search strategy for at least 1 database is provided as a Multimedia Appendix 1. The third step involved a supplementary search, which included a manual review of the reference lists of all included studies to identify other relevant papers not captured by the database searches. The search was limited to papers published between January 2015 and June 2026 to ensure relevance to contemporary technological advancements. No language restrictions were applied to the search. The PubMed search strategy is detailed in Textbox 2.

Textbox 2. **PubMed search strategy.**

“Hypertension”[Mesh] OR “Blood Pressure”[Mesh] OR hypertens*[tiab] OR “high blood pressure”[tiab] OR “elevated blood pressure”[tiab] OR “raised blood pressure”[tiab] OR “uncontrolled blood pressure”[tiab] OR “blood pressure control”[tiab] OR “blood pressure management”[tiab] OR “BP control”[tiab] OR “BP management”[tiab] OR “essential hypertension”[tiab] OR “primary hypertension”[tiab] OR “uncontrolled hypertension”[tiab] OR “resistant hypertension”[tiab] OR “arterial hypertension”[tiab] OR “hypertensive patient*”[tiab] OR “hypertensive individual*”[tiab] OR “hypertensive adult*”[tiab] OR “systolic hypertension”[tiab] OR “diastolic hypertension”[tiab] OR antihypertens*[tiab] OR “lowering blood pressure”[tiab] OR “high BP”[tiab]

AND

“Artificial Intelligence”[Mesh] OR “Machine Learning”[Mesh] OR “Deep Learning”[Mesh] OR “Natural Language Processing”[Mesh] OR “Neural Networks, Computer”[Mesh] OR “Decision Support Systems, Clinical”[Mesh] OR “Expert Systems”[Mesh] OR “artificial intelligence”[tiab] OR AI[tiab] OR “machine learning”[tiab] OR “deep learning”[tiab] OR “neural network*”[tiab] OR “natural language processing”[tiab] OR NLP[tiab] OR “large language model*”[tiab] OR LLM[tiab] OR LLMs[tiab] OR “LLM-based”[tiab] OR “LLM-driven”[tiab] OR ChatGPT[tiab] OR GPT[tiab] OR “generative AI”[tiab] OR “generative artificial intelligence”[tiab] OR “generative pretrained transformer*”[tiab] OR “expert system*”[tiab] OR “knowledge graph*”[tiab] OR “knowledge base*”[tiab] OR “clinical decision support*”[tiab] OR “decision support system*”[tiab] OR “chatbot*”[tiab] OR “chat-bot*”[tiab] OR “conversational agent*”[tiab] OR “virtual assistant*”[tiab] OR “intelligent system*”[tiab] OR “recommender system*”[tiab] OR “predictive model*”[tiab] OR “prediction model*”[tiab] OR “random forest*”[tiab] OR “support vector machine*”[tiab] OR SVM[tiab] OR “reinforcement learning”[tiab] OR “transformer model*”[tiab] OR BERT[tiab] OR “bidirectional encoder”[tiab] OR “retrieval-augmented generation”[tiab] OR RAG[tiab] OR “text mining”[tiab] OR “speech recognition”[tiab] OR “fuzzy logic”[tiab] OR “Bayesian network*”[tiab] OR “ontology”[tiab] OR “supervised learning”[tiab] OR “unsupervised learning”[tiab] OR “data mining”[tiab] OR “pattern recognition”[tiab] OR “computational intelligence”[tiab]

AND

“Health Education”[Mesh] OR “Patient Education as Topic”[Mesh] OR “Self Care”[Mesh] OR “Self-Management”[Mesh] OR “Patient Compliance”[Mesh] OR “Health Promotion”[Mesh] OR “Health Communication”[Mesh] OR “Health Literacy”[Mesh] OR “health education”[tiab] OR “patient education”[tiab] OR “health promotion”[tiab] OR “health communication”[tiab] OR “health information”[tiab] OR “patient information”[tiab] OR “patient teaching”[tiab] OR “patient counselling”[tiab] OR “patient counseling”[tiab] OR “self-management”[tiab] OR “self-management education”[tiab] OR “self care”[tiab] OR “self-care”[tiab] OR “lifestyle modification*”[tiab] OR “lifestyle intervention*”[tiab] OR “lifestyle change*”[tiab] OR “behavioral intervention*”[tiab] OR “behavioural intervention*”[tiab] OR “behavior change”[tiab] OR “behaviour change”[tiab] OR “health coaching”[tiab] OR “medication adherence”[tiab] OR “treatment adherence”[tiab] OR “therapeutic adherence”[tiab] OR “patient adherence”[tiab] OR “patient empowerment”[tiab] OR “health knowledge”[tiab] OR “patient knowledge”[tiab] OR “patient engagement”[tiab] OR “educational intervention*”[tiab] OR “educational program*”[tiab] OR “educational material*”[tiab] OR “health behavior”[tiab] OR “health behaviour”[tiab] OR “dietary advice”[tiab] OR “dietary education”[tiab] OR “exercise counseling”[tiab] OR “exercise education”[tiab] OR “lifestyle guidance”[tiab] OR “lifestyle advice”[tiab] OR “health advice”[tiab] OR “consumer health information”[tiab]

### Selection of Sources of Evidence

After the search was completed, all retrieved records were organized and imported into EndNote (Clarivate), and duplicate entries were removed. Two independent reviewers (HC and SX) screened the titles and abstracts to assess whether they met the inclusion criteria. For potentially relevant papers, the same 2 independent reviewers retrieved the full texts and conducted a detailed assessment based on the inclusion criteria. The reasons for excluding full-text papers that did not meet the inclusion criteria were documented and presented in the PRISMA flowchart. In accordance with guidelines for supplementary searches, the reference lists of all included studies were manually searched to identify other relevant literature cited in the included studies. Records identified through these supplementary searches underwent the same screening process described earlier. At each stage of the screening process, any disagreements among reviewers were resolved through discussion or with the assistance of a third reviewer (ZW). The search results and the study screening process have been fully reported in the PRISMA-ScR flowchart.

### Data Charting Process

Two independent reviewers (HC and SX) extracted data from the included studies using a standardized data extraction form developed in Microsoft Excel. The 2 reviewers pilot-tested the form on 3 randomly selected included studies and refined it before proceeding with the full data extraction. Disagreements that arose during data extraction were resolved through discussion or by consulting a third reviewer (ZW). When necessary, the corresponding authors of the included studies were contacted to request missing or supplementary data.

### Data Items

The following data items were extracted from each included study:

- Basic information: First author, year of publication, and country.
- Study design and methods: Research design classified via Mixed Methods Appraisal Tool (MMAT) categories, study setting, sample size, and duration.
- AI technical characteristics: AI technology type, specific AI technology or model name, core AI techniques used, application scenario, and brief description of application scenario.
- Characteristics of health education: Health education content or domains.
- Outcome measures: All outcome measures reported by the authors and summary of reported conclusions.

### Critical Appraisal of Individual Sources of Evidence

No formal critical appraisal of individual evidence sources was undertaken. As a scoping review, this study was designed to map the breadth and characteristics of the available evidence. Because of the heterogeneity in study designs and the exploratory nature of the field, all eligible primary studies were retained for data charting and narrative synthesis. The absence of formal critical appraisal is acknowledged as a limitation and is taken into consideration when interpreting the findings.

### Synthesis of Results

Charted data were synthesized using narrative synthesis and organized by AI technology type, application scenario, health education characteristics, and outcome measures. Findings were summarized using tables, figures, and an evidence gap map.

## Results

### Selection of Sources of Evidence

**Figure 1.**
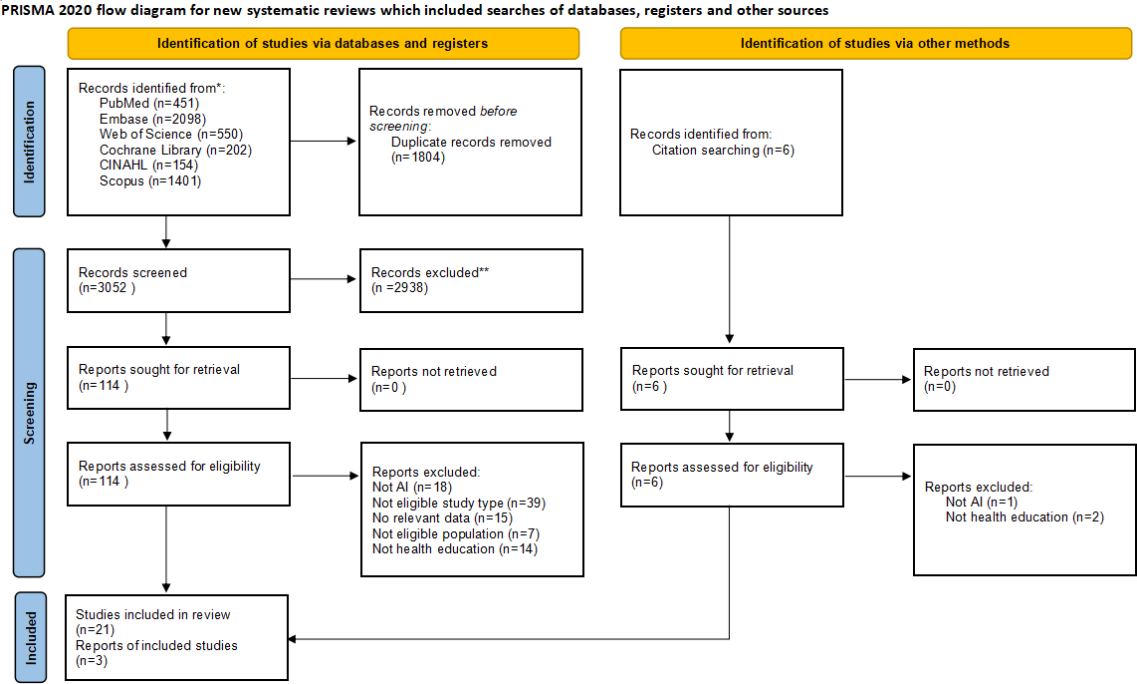
PRISMA (Preferred Reporting Items for Systematic Reviews and Meta-Analyses) flow diagram for study selection process for a scoping review of application of artificial intelligence in hypertension health education (2020‐2026) (reproduced from Page et al [30], which is published under Creative Commons Attribution 4.0 International License [31]). AI: artificial intelligence.

The study screening process is summarized in the PRISMA flowchart (Figure 1). A total of 4856 records were retrieved from the databases. Among these, 1804 were duplicates, leaving 3052 records to proceed to the screening stage. Of these, 2938 records were excluded because they did not meet the inclusion criteria. A total of 114 studies were downloaded for full-text screening. Following full-text review, 21 studies were included. Through citation searching, an additional 6 records were identified from these studies, of which 3 were ultimately included. This review ultimately included 24 papers sourced from databases and reference lists.

### Characteristics of Sources of Evidence

**Figure 2.**
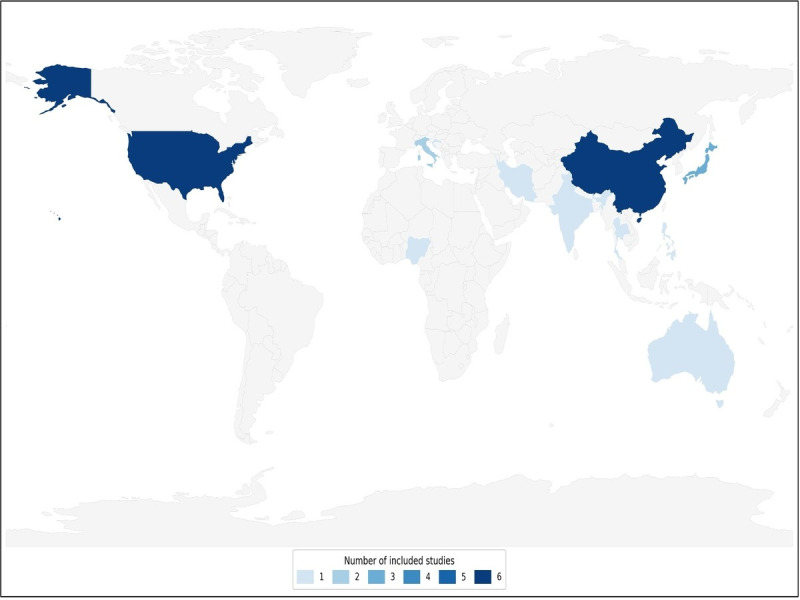
Included studies by the number of publications from the country of the first author.

This review included a total of 24 studies published between 2020 and 2026. The majority of these studies (17/24, 71%) were published between 2024 and 2026, while studies published before 2022 accounted for only 13% (3/24). These studies spanned 11 countries, with China and the United States each accounting for 25% (6/24), and Japan accounting for 13% (3/24). Additionally, 5 studies were conducted in low- and middle-income countries (Philippines, India, Iran, Nigeria, and Thailand). Figure 2 summarizes the distribution of the 24 included studies by the first author’s country of affiliation.

### Critical Appraisal Within Sources of Evidence

No formal critical appraisal of individual evidence sources was undertaken. As a scoping review, this study was designed to map the breadth and characteristics of the available evidence.

### Results of Individual Sources of Evidence

We classified the study designs of each study based on the MMAT framework [32] and summarized the methodologies used. It should be noted that the MMAT is typically used to assess the quality of mixed methods research. However, in this scoping review, we used only its study design classification framework to categorize the literature and did not conduct a formal quality assessment. This approach aligns with the methodological principles of scoping reviews, which aim to map the evidence landscape within a specific field rather than critically assess the quality of included studies [25,33]. Among the 24 included studies, quantitative descriptive studies constituted the largest proportion (n=11, 46%), followed by randomized controlled trials (RCTs; n=6, 25%), quantitative nonrandomized trials (n=4, 17%), and mixed methods studies (n=3, 13%). The main characteristics of the studies included in this review are presented in Table 1. A summary table of characteristics compiled from the data of the included studies can be found in Multimedia Appendix 2 [34-57].

**Table 1.** The main characteristics of the studies included in author, country, research design, sample size and groups, and duration.

Author (year)	Country	Research design	Sample size and groups	Duration
Persell et al (2020) [34]	United States	Quantitative RCT^a^	n=333 randomized (intervention=166, control=167) n=297 completed follow-up	6 months
Griffin et al (2021) [35]	United States	Mixed methods study	n=15 adults with hypertension prescribed ≥1 medication	Single time point
Kario et al (2021) [36]	Japan	Quantitative RCT	n=390 randomized (digital therapeutics=199, control=191) n=372 completed 12-week follow-up	24 weeks
Gutierrez and Sakulbumrungsil (2023) [37]	Philippines	Quantitative RCT	n=417 randomized (intervention=214, control=203) n=401 completed 6-month follow-up	6 months
Griffin et al (2023) [38]	United States	Quantitative descriptive study	n=10 patients with hypertension	Single time point
Sakane et al (2023) [39]	Japan	Quantitative RCT	n=78 men who are overweight or obese aged 40‐69 years with hypertension (intervention=39, control=39) Retention=95%	12 weeks
Yano et al (2024) [40]	Japan	Quantitative descriptive study	20 questions generated by ChatGPT Evaluated by 3 blinded certified hypertension or nephrology specialists	Single time point
O’Hagan et al (2023) [41]	Australia	Quantitative descriptive study	15 hypertension-related questions (2 separate question sets with different wording) Evaluated by 2 independent reviewers on 2 separate occasions	Three evaluation time points: February, April, and May 2023
Almagazzachi et al (2024) [42]	United States	Quantitative descriptive study	100 questions, each posed to ChatGPT 3 times by 3 different study staff Reviewed by board-certified internal medicine physician	Single evaluation period
Lee et al (2024) [43]	United States	Quantitative descriptive study	52 ACC^b^ frequently asked questions on hypertension 2 AI^c^ chatbots (ChatGPT 3.5 and Gemini 1.0) 4 prompt forms (no prompt, patient-friendly, physician-level, and statistics or references) Total 416 responses analyzed	Single evaluation period
Vinufrancis et al (2024) [44]	India	Quantitative descriptive study	10 common patient queries about hypertension 2 AI chatbots (ChatGPT and ChatSonic) 2 internal medicine physician evaluators Responses assessed per query	Single evaluation period
Leitner et al (2024) [45]	United States	Quantitative nonrandomized trial	n=141 adults with hypertension n=128 at 12 weeks n=102 at 24 weeks	24 weeks
Niko et al (2024) [46]	Iran	Quantitative descriptive study	10 HBPM^d^ questions, answered twice per chatbot Evaluated by 3 certified cardiologists	Single evaluation period
Sun et al (2024) [47]	China	Quantitative RCT	n=68 enrolled, randomized 1:1 n=54 analyzed (experimental=23, control=31)	12 weeks
Xu et al (2024) [48]	China	Mixed methods study	5 patients with hypertension comorbidities 24 multidisciplinary experts evaluated	Single evaluation period (December 2023 to February 2024)
Aguzzi et al (2025) [49]	Italy	Quantitative descriptive study	21 QA^e^ test pairs (from augmented dataset of 1473 records) Multiple RAG^f^ strategies compared, domain expert evaluation	Single evaluation period
Antia et al (2025) [50]	Nigeria	Quantitative nonrandomized trial	n=50 adults on hypertension treatment n=48 completed follow-up 2 (4%) lost to follow-up	30 days
Jelic et al (2025) [51]	Croatia	Mixed methods study	n=5136 users of Megi (an AI-based digital health tool for hypertension management) n=125 users analyzed from online survey of Megi	24 months (for quantitative data) Single survey time point (for qualitative data)
Montagna et al (2025) [52]	Italy	Quantitative descriptive study	Simulated patient query dataset 128 questions for BERT^g^ score 210 responses evaluated by domain experts	Single evaluation period
Moolsart and Kritpolviman (2025) [53]	Thailand	Quantitative nonrandomized trial	n=91 older adults aged 60‐75 years with uncontrolled hypertension (experimental=46, comparison=45)	12 weeks
Wang et al (2025) [54]	China	Quantitative nonrandomized trial	n=51 patients with hypertension from 3 centers n=6 clinicians 10 FAQ^h^ for BP^i^ Coach comparison	30 days
Wang et al (2026) [55]	China	Quantitative descriptive study	50 hypertension-related questions across 4 domains 8 model configurations (4 base+4 HEART^j^-enhanced) 3 clinicians blind evaluation	Single evaluation period
Wang et al (2026) [56]	China	Quantitative descriptive study	Internal testing: BKQA^k^ (n=20), BPQA^l^ (n=200), RFQA^m^ (n=139), DMQA^n^ (n=200) External testing: n=107 suspected patients with hypertension Benchmarked against 3 physicians	Internal: August 2024 to February 2025 External: February 2025 to June 2025
Yao et al (2026) [57]	China	Quantitative RCT	n=62 patients with hypertension (intervention=31, control=31) CPET^o^ subsample n=24	8 weeks

a RCT: randomized controlled trial.

b ACC: American College of Cardiology

c AI: artificial intelligence.

d HBPM: home blood pressure monitoring.

e QA: question answering.

f RAG: retrieval-augmented generation.

g BERT: Bidirectional Encoder Representation from Transformers.

h FAQ: frequently asked question.

i BP: blood pressure.

j HEART: Hypertension Enhancing Answer Retrieval Tool.

k BKQA: blood pressure knowledge question and answer.

l BPQA: blood pressure question and answer.

m RFQA: risk factor question and answer.

n DMQA: decision-making question and answer.

o CPET: cardiopulmonary exercise testing.

### Synthesis of Results

#### Overview

**Figure 3.**
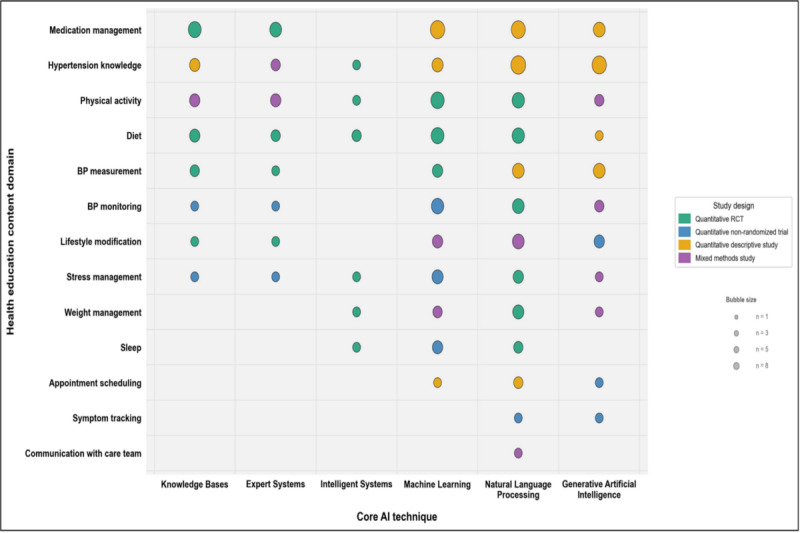
Evidence gap map of core AI technologies used in hypertension health education across the 24 included studies. AI: artificial intelligence; BP: blood pressure; RCT: randomized controlled trial.

In this study, we analyzed data from the 24 included studies and created an evidence gap map (Figure 3) to illustrate the core AI technologies used in each study.

Among the included studies, NLP was the most widely used technology; nearly all studies involved text or dialogue processing for knowledge transfer, interactive learning, and behavioral guidance in hypertension health education. For example, rule-based dialogue agents and virtual nurse chatbots are typical applications of NLP technology, providing patients with personalized education and self-management support [34,36].

The introduction of generative AI marks a new development in this field, with its applications increasing significantly since 2023. In particular, the emergence of LLMs, such as the study by O’Hagan et al [41], which first evaluated the application of ChatGPT in hypertension health education, has led to a rapid increase in the number of LLM-related studies since 2024. This indicates that generative AI holds potential for improving educational interactions, personalizing information, and generating content.

Regarding the distribution of technology categories, expert system and knowledge base remain prevalent, indicating that traditional AI methods still hold value in the management of structured educational content and knowledge transfer. Furthermore, NLP and ML technologies are frequently applied together in many studies, suggesting that text processing and algorithm-driven intelligent assistance are jointly supporting the implementation of hypertension health education.

The evidence gap map reveals that, despite the widespread application of NLP and ML technologies, the use of generative AI is still in its infancy. The integration of specific AI technologies with certain areas of health education, such as symptom tracking, clinician-patient communication, and appointment management, remains relatively scarce, providing a clear direction for future research. Overall, AI applications in the field of hypertension health education are rapidly evolving from rule-based and traditional algorithms toward generative and interactive technologies.

**Figure 4.**
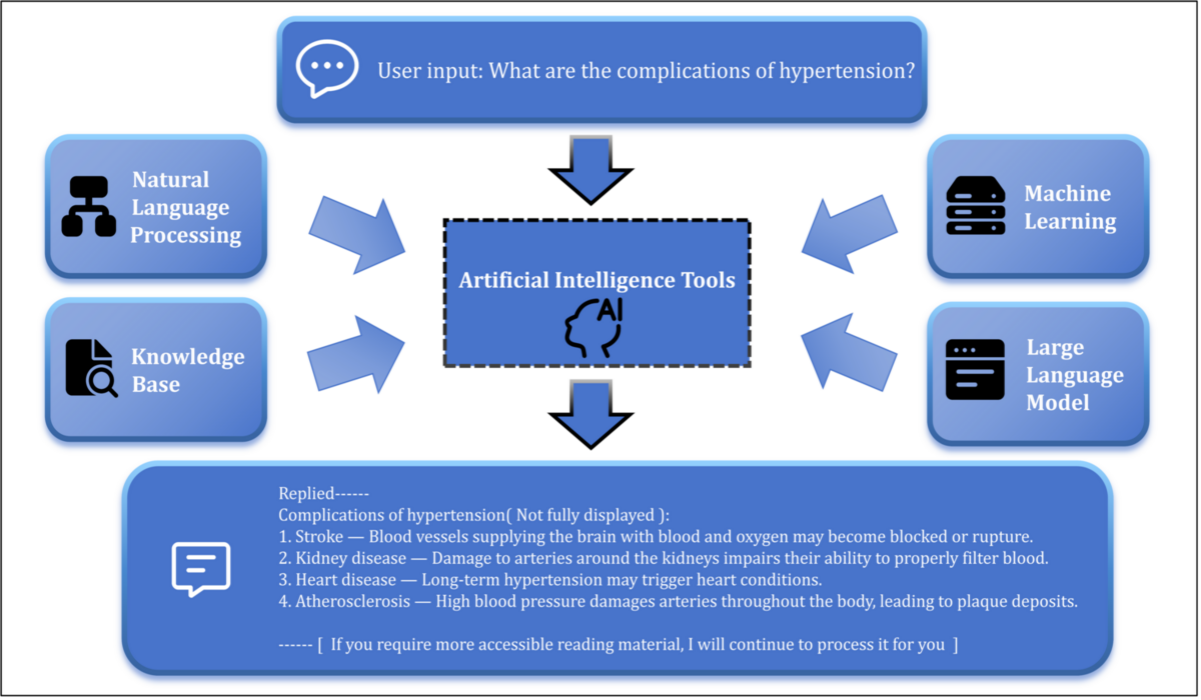
A conceptual model for an artificial intelligence–based hypertension health education system.

As shown in Figure 4, we demonstrate how AI technology is applied to health education for hypertension.

#### Application Scenario of AI

Based on data extracted from 24 studies, we have categorized the applications of AI in hypertension health education into three types (Tables2  3): (1) rule-based health education, (2) data-driven adaptive health education, and (3) generative AI–driven health education. Among these, rule-based health education and generative AI–driven health education are the most common. Rule-based health education is characterized by content determined by predefined rules and knowledge structures, thereby offering determinism and interpretability. Data-driven adaptive health education uses ML models trained on user behavior and physiological data to provide personalized intervention recommendations or real-time feedback to individuals while adaptively adjusting educational content. Generative AI–driven health education leverages LLMs, combined with techniques, such as retrieval-augmented generation (RAG) and intelligent agents, to support open-ended question-and-answering and personalized dialogue, thereby delivering health education. It is important to distinguish between studies that evaluated LLM outputs against reference standards (proof-of-concept accuracy assessments) and those that deployed LLM-based tools in interactive educational settings with real patients; the majority of generative AI studies in this review fall into the former category, reflecting the early developmental stage of this application scenario.

**Table 2.** Classification and comparative characteristics of AI application scenarios in hypertension health education.

Application scenario	Technical foundation	Content determination method	Adaptability	Interaction
Rule-based health education	ES^a^, KB^b^, decision trees	Predefined rules and knowledge structures	Low, fixed paths	Push of fixed graphics or texts, structured courses
Data-driven adaptive health education	Traditional ML^c^ (eg, collaborative filtering and reinforcement learning)	User behavior and physiological data models	Medium, dynamically adjusts with data	Personalized recommendations, real-time feedback reminders
Generative AI^d^-driven health education	LLM^e^+RAG^f^ or agent	Generative dynamic synthesis	High, open-domain Q&A^g^	Multiturn dialogue, natural language Q&A

a ES: expert system.

b KB: knowledge base.

c ML: machine learning.

d AI: artificial intelligence.

e LLM: large language model.

f RAG: retrieval-augmented generation.

g Q&A: question and answer.

**Table 3.** Distribution of the 24 included studies by artificial intelligence (AI) application scenario.

Application scenario	Studies	Year range	Study design
Rule-based health education	[34-39,47,51]	2020‐2025	Quantitative RCT^a^ (n=4) Mixed methods study (n=1) Quantitative descriptive study (n=2) Quantitative nonrandomized trial (n=1)
Data-driven adaptive health education	[45,53,57]	2024‐2026	Quantitative nonrandomized trial (n=2) Quantitative RCT (n=1)
Generative AI-driven health education	[40-44,46,48-50,52,54-56]	2023‐2026	Quantitative descriptive study (n=10) Quantitative nonrandomized trial (n=1) Mixed methods study (n=2)

a RCT: randomized controlled trial.

These 24 studies were categorized into 3 distinct application scenarios and exhibited clear patterns in terms of both time span and research methods. Rule-based systems (n=8) spanned the entire review period (2020‐2025) and accounted for 4 of the 6 RCTs. Data-driven adaptive interventions (n=3) began to emerge in 2024, primarily using nonrandomized designs. Generative AI–driven applications (n=13) have dominated the literature since 2023 but remain concentrated in the proof-of-concept phase. In total, 8 of the 11 studies used quantitative descriptive designs, and none were evaluated via RCTs.

#### Characteristics of Health Education

We summarized the characteristics of health education based on data from the included studies, as shown in Table 4.

**Table 4.** Characteristics of health education: author, health education content or domains, and its role in study.

Author (year)	Health education content or domains	The role of health education in study
Persell et al (2020) [34]	BP^a^ self-monitoring Medication adherence Weight loss Diet Physical activity Sleep Stress management	As a component integrated with home blood pressure monitoring (HBPM) and medication tracking to enable interventions
Griffin et al (2021) [35]	Medication management Medication refills Communication with care team Healthy lifestyles (diet, exercise, and BP tracking)	Providing health education content to support self-management of hypertension
Kario et al (2021) [36]	Salt restriction Body weight control Regular exercise Improving sleep condition Stress coping Reducing alcohol intake BP management knowledge	As a part of a digital therapeutics system
Gutierrez and Sakulbumrungsil (2023) [37]	Medication knowledge (reason for taking, side effects, duration, and monitoring) BP monitoring; lifestyle modifications What to do if dose missed Proper medication storage	As a part of a pharmacist-led expert system
Griffin et al (2023) [38]	Medication information (list, reminders, and side effects) Medication refills Healthy recipes Appointment scheduling BP tracking and sharing with providers	Providing health education content to support self-management of hypertension
Sakane et al (2023) [39]	Healthy eating habits Exercise habits Lifestyle habits BP monitoring Daily steps (5000/7000/8000/10,000 targets) Weight management	As a part of a smartphone weight-loss app
Yano et al (2024) [40]	All kinds of hypertension information produced due to the inquiries	Large language models provide health education information
O’Hagan et al (2023) [41]	All kinds of hypertension information produced due to the questions	Large language models provide health education information
Almagazzachi et al (2024) [42]	All kinds of hypertension information produced due to the questions	Large language models provide health education information
Lee et al (2024) [43]	All kinds of hypertension information produced due to the questions	Large language models provide health education information
Vinufrancis et al (2024) [44]	All kinds of hypertension information produced due to the questions	Large language models provide health education information
Leitner et al (2024) [45]	Physical activity (steps and active time) Sleep hygiene Stress management Dietary choices (salt, alcohol, red meat, and fruits or vegetables) Medication adherence BP self-monitoring	As a part of an artificial intelligence (AI)–powered lifestyle coaching program
Niko et al (2024) [46]	Self-preparation before measurement (clothing, rest, and avoidance of stimuli) Body position and cuff use Number of measurement repetitions Correct recording and reading of HBPM	Large language models provide health education information
Sun et al (2024) [47]	Exercise prescriptions (frequency, intensity, type, time, volume, and progression) DASH^b^ diet (types and quantities of food) Medication adherence BP monitoring frequency and technique	As part of a smart health promotion system based on the WeChat platform
Xu et al (2024) [48]	Exercise prescription (expected health benefits, exercise principles—FITT^c^, weekly plan, movement guidance, and precautions) Disease-specific education (hypertension, diabetes, COPD^d^, Parkinson, gout, and chronic nephritis)	Large language models provide health education information
Aguzzi et al (2025) [49]	All kinds of hypertension knowledge: BP monitoring, lifestyle modifications, medication management	Large language models provide health education information
Antia et al (2025) [50]	Hypertension education (disease knowledge and risk factors) Medication adherence reminders Clinic appointment reminders Symptom tracking Lifestyle modification guidance	As a part of a WhatsApp-based generative AI chatbot
Jelic et al (2025) [51]	BP monitoring Medication adherence Physical activity Weight management Lifestyle modification Stress reduction	As a part of a chatbot based on a large language model
Montagna et al (2025) [52]	Hypertension general knowledge	Large language models provide health education information
Moolsart and Kritpolviman (2025) [53]	BP self-monitoring skills Dietary management (food tracking with carbohydrate or protein or fat or sugar or sodium details) Physical activity (step counting and exercise) Stress management (relaxation breathing and positive thinking) Medication adherence	As a part of an AI-based self-health monitoring program
Wang et al (2025) [54]	BP assessment Behavior change (lifestyle habits, exercise, diet, sleep, and mental health) Digital phenotyping education Medication management; treatment reminders	As a part of a multimodal digital platform for hypertension management
Wang et al (2026) [55]	BP knowledge (systolic or diastolic, normal values, diagnosis, classification, risk factors, and complications) BP measurement (methods, cuff selection, timing, frequency, and correct technique) BP management (diet, fruits or vegetables, alcohol, weight, exercise, sauna, stress, sleep, and e-cigarettes) Medication management (necessity, tolerance, organ damage, timing, and drug interactions)	Large language models provide health education information
Wang et al (2026) [56]	Hypertension knowledge	Large language models provide health education information
Yao et al (2026) [57]	Exercise prescription (warm-up, cardiorespiratory endurance training, strength resistance training, balance training, flexibility training, and cooldown) Exercise safety and posture correction Lifestyle guidance	As a part of an AI-assisted CPET^e^ exercise prescription tool

a BP: blood pressure.

b DASH: dietary approaches to stop hypertension.

c FITT: Frequency, Intensity, Time, Type.

d COPD: chronic obstructive pulmonary disease.

e CPET: cardiopulmonary exercise testing.

#### Outcomes Measured to Evaluate AI-Based Hypertension Health Education

After analyzing the included studies, we found that their primary research objectives could be broadly categorized into 2 types: one aimed to evaluate the clinical efficacy of AI-based hypertension health education interventions, while the other assessed the performance of LLMs or AI-based hypertension health education systems. Studies in the first category reported outcomes related to clinically relevant measures. The second category of studies involved the measurement of common computer-related metrics, such as system usability, the accuracy of health education information, and readability. The outcome characteristics of the included studies are shown in Table 5.

**Table 5.** Outcome measures of included studies.

Author (year)	Outcome measures
Persell et al (2020) [34]	Primary: SBP^a^ at 6 months Secondary: Self-reported antihypertensive medication adherence, home monitoring and self-management practices, measures of self-efficacy associated with blood pressure (BP), weight, and self-reported health behaviors
Griffin et al (2021) [35]	Information-need themes: Perceptions toward chatbots Perceived frequency of use Barriers and facilitators of using
Kario et al (2021) [36]	Primary: Mean change in 24-hour ambulatory SBP from baseline to 12 weeks Secondary: Home SBP or DBP^b^ (morning and evening), office SBP or DBP, heart rate, salt intake, body weight, BMI, app engagement rate, self-reported executive scores for app-guided behaviors, and adverse events
Gutierrez and Sakulbumrungsil (2023) [37]	Primary: Medication Possession Ratio Secondary: Medication adherence, SBP, DBP, controlled BP, number of antihypertensive agents, medication changes, and self-perceived knowledge score
Griffin et al (2023) [38]	Effectiveness: Task completion rate, user error rate, and system error rate Efficiency: Number of clicks, utterances, and duration of interaction per task Satisfaction: System Usability Scale Qualitative feedback (strengths and shortcomings)
Sakane et al (2023) [39]	Body weight, BMI, SBP, DBP, adherence to daily self-weighing, pedometer use, BP monitoring, self-reported health behaviors (exercise habits, eating habits, lifestyle habits, and daily steps), and personality traits
Yano et al (2024) [40]	Appropriateness of responses Comparison of Japanese versus English response quality (accuracy, comprehensiveness, professionalism, and level of detail) Gwet agreement coefficient for interrater reliability
O’Hagan et al (2023) [41]	Readability Credibility Accuracy
Almagazzachi et al (2024) [42]	Accuracy Reproducibility
Lee et al (2024) [43]	Proportions of correct, partially correct, and incorrect responses Flesch-Kincaid grade level Word count
Vinufrancis et al (2024) [44]	Global Quality Scale scores Modified DISCERN scores Cohen κ for interrater agreement
Leitner et al (2024) [45]	Primary: SBP and DBP change from baseline to 12 and 24 weeks and percentage change in BP categories (controlled, stage-1, and stage-2) Secondary: Participant engagement rate and number of manual clinician outreaches
Niko et al (2024) [46]	Accuracy Completeness Reproducibility
Sun et al (2024) [47]	Primary: SBP, DBP, exercise time, medication adherence, DASH^s^ adherence, and BP monitoring frequency Secondary: Weight, SEVR^d^, baPWV^e^, heart rate, learning performance, diet types or quantity, and weekly adherence curves
Xu et al (2024) [48]	Accuracy, comprehensiveness, and applicability Qualitative narrative recommendations from 24 multidisciplinary experts Kendall concordance coefficients for expert agreement
Aguzzi et al (2025) [49]	Faithfulness Medical faithfulness Domain expert evaluation scores Chi-square tests with Cramér *V* for RAG^f^ versus non-RAG comparison
Antia et al (2025) [50]	Feasibility: Training time (minutes), proportion able to use bot within 5 minutes Acceptability: frequency of chats, Chatbot Usability Questionnaire, and Self-made Healthy Heart Assistant Satisfaction Questionnaire Preliminary efficacy: SBP or DBP, hypertension knowledge test, and medication adherence
Jelic et al (2025) [51]	User retention at 3, 6, 9, 12, and 24 months SBP reduction Spearman correlation between SBP drop and duration of use Online survey: Usefulness in improving self-management, satisfaction, willingness to use, behavioral response, and unmet needs
Montagna et al (2025) [52]	Intent recognition: Precision, recall, and accuracy per class and overall Data extraction accuracy (measure, quantity, format, and overall) BERT^g^ score (precision, recall, and *F*_1_ vs GPT-3.5 Turbo reference) Domain expert evaluation Chatbot response time
Moolsart and Kritpolviman (2025) [53]	Primary: Hypertension-controlling behavior Secondary: mean arterial pressure, SBP, and DBP
Wang et al (2025) [54]	Patient: MAUQ^h^ subscales—ease of use and satisfaction, system information arrangement, and usefulness Clinician: Doctor’s Software Satisfaction Questionnaire and patient management time BP coach: Utility, conciseness, completeness, and clarity
Wang et al (2026) [55]	Accuracy, completeness, consistency, robustness, and security Overall quality Interrater reliability Cohen *d* effect sizes Between-group comparisons (base vs HEART^i^-enhanced)
Wang et al (2026) [56]	Internal testing: BP classification accuracy, risk factor stratification accuracy, clinical decision appropriateness, education comprehensiveness and accuracy, discriminative performance (AUC^j^), decision curve analysis, IDI^k^, and NRI^l^ External validation: BP classification accuracy, risk stratification accuracy, clinical decision accuracy, patient-perceived understandability, credibility, and emotional support
Yao et al (2026) [57]	Primary: 6-minute walk distance Secondary: SBP, DBP, CPET^m^ indices (peak VO₂ [mL/min/kg], peak VO₂%pred [%], total exercise time, anaerobic threshold, RER^n^, maximum load, and heart rate at rest per peak), IPAQ^o^, SF-12^p^, PHQ-9^q^, GAD-7^r^, exercise self-efficacy, body weight, handgrip strength (right or left), and waist or hip circumference

a SBP: systolic blood pressure.

b DBP: diastolic blood pressure.

c DASH: dietary approaches to stop hypertension.

d SEVR: subendocardial viability ratio.

e baPWV: brachial-ankle pulse wave velocity.

f RAG: retrieval-augmented generation.

g BERT: Bidirectional Encoder Representation from Transformers.

h MAUQ: mHealth App Usability Questionnaire.

i HEART: Hypertension Enhancing Answer Retrieval Tool.

j AUC: area under the curve.

k IDI: integrated discrimination improvement.

l NRI: Net Reclassification Index.

m CPET: cardiopulmonary exercise testing.

n RER: respiratory exchange ratio.

o IPAQ: International Physical Activity Questionnaire.

p SF-12: 12-item Short Form Health Survey.

q PHQ-9: Patient Health Questionnaire-9.

r GAD-7: Generalized Anxiety Disorder-7.

We categorized all studies according to the definitions in the Digital Health Scorecard Framework [58]. The Digital Health Scorecard Framework encompasses 4 domains: technical, clinical, usability, and cost. In this framework, technical refers to evaluating whether a digital health solution can accurately and precisely deliver its claimed functionality, including considerations such as security, interoperability, and system architecture. The clinical dimension focuses on rigorous evaluation of evidence to validate whether the solution has demonstrated capacity to improve specific health outcomes, requiring comparison against relevant clinical gold standards; usability concerns the ease with which users can accomplish intended tasks with minimal effort, encompassing aspects like effectiveness, learnability, and user satisfaction; and cost includes user access fees, technology life-cycle investments, and integration expenses within clinical workflows.

#### Technical

In the included studies, accuracy was the most frequently evaluated metric. The performance of AI-based hypertension health education systems needs to be compared against reference standards, such as clinical guidelines, medical textbooks, and expert-developed question sets [40-43,46,48,49,52,55,56]. The completeness and comprehensiveness of health education content are also key metrics [46,48,55,56]. In addition, readability and credibility were evaluated [41], both of which are related to the accuracy and reliability of information delivery. One study further assessed readability using objective measures including the Flesch-Kincaid grade level and response length, providing additional evidence regarding the accessibility of AI-generated educational content [43].

The system’s performance in the face of external disturbances is reflected in the assessment of robustness when handling unexpected inputs [55]. According to the framework, privacy and security are explicitly listed as part of the technical evaluation, which aligns with the current requirement that AI applications in medicine must protect patient data [55]. Other relevant technical metrics include fidelity and medical fidelity [49], intent recognition and data extraction accuracy [52], applicability [48], and the appropriateness and professionalism of responses. Vinufrancis et al [44] additionally evaluated information quality and reliability using the Global Quality Scale and the modified DISCERN instrument, highlighting the importance of assessing the educational value and trustworthiness of AI-generated health information. Domain expert evaluations [49,52] and interrater reliability [40,44,55] serve as complementary methods for validating technical performance.

#### Clinical

Blood pressure, as a key clinical end point, has been reported in numerous studies, with measurement methods including office blood pressure, home blood pressure, and 24-hour ambulatory blood pressure monitoring [34,36,37,39,45,47,50,51,53,57]. This underscores the need to compare any AI system used in clinical practice against established clinical gold standards. Other cardiovascular-related parameters, such as heart rate [36,47], subendocardial viability ratio, and brachial-ankle pulse wave velocity [47], as well as cardiopulmonary exercise test parameters and 6-minute walk distance [57], were also included in the assessment of clinical outcomes.

The process indicators emphasized by this framework are particularly evident in the assessment of medication adherence, specifically treatment adherence or adherence to clinical guidelines. The indicators used include medication possession rates, self-reported adherence, and the Morisky Medication Adherence Scale [34,37,47,50]. The number of antihypertensive medications and medication adjustments [37] are also used as process-related indicators. In one study [53], blood pressure control behaviors served as the primary outcome measure, and behavioral monitoring was reported—including blood pressure monitoring frequency, daily self-weighing, pedometer use, and dietary approaches to stop hypertension diet adherence [39,47]. Self-reported health behaviors [34,39] and salt intake [36] also meet the definition of process measures.

Other relevant clinical indicators include self-efficacy related to blood pressure [34] and physical activity [57], as well as knowledge about hypertension [37,47,50], academic performance [47], weight, BMI [34,36,39,47,57], waist, hip circumference [57], grip strength [57], mental health scales [57], and app-guided behavioral adherence scores [36].

#### Cost

Among the included studies, cost assessment was the least well-developed dimension, which is consistent with the framework’s observation that comprehensive cost estimation is a complex process. None of the studies included in this review reported formal cost-effectiveness analyses, technology lifecycle costs, or the long-term economic impacts of clinical efficacy improvements.

#### Usability

The included studies used various methods to assess usability. Griffin et al [38] used the System Usability Scale to capture users’ subjective evaluations of ease of use and likability. Task-level efficiency was measured by task completion rates, user error rates, number of clicks, number of voice commands, and interaction duration per task [38]. Chatbot response times [52] and qualitative feedback regarding their strengths and weaknesses were used to assess user experience. Two additional studies also assessed user experience using the “Chatbot Usability Questionnaire” and a custom-designed satisfaction questionnaire [50], combined with online surveys covering dimensions such as practicality, satisfaction, willingness to use, behavioral responses, and unmet needs [51].

Patient engagement is also a key indicator of usability, with metrics including user retention rates over a 24-month period [51], chat frequency [50], and app engagement rates [36,45]. For example, training time and the proportion of users able to operate the chatbot within 5 minutes [50] are also used to assess usability. Explorations of chatbot cognition, perceived use frequency, and barriers and facilitators [35] provide insights into user needs, reflecting the necessity of user-centered design for AI systems.

Usability for clinicians was assessed using the Physician Software Satisfaction Questionnaire and patient management time [54], indicating that AI-based hypertension health education systems should not increase the burden on clinical staff. The patient-oriented Mobile Health App Usability Questionnaire covered ease of use, satisfaction, system information layout, and practicality [54], while also incorporating patient-perceived comprehensibility, credibility, and emotional support [56], thereby reflecting usability at the subjective level. The evaluation of BP Coach in terms of practicality, simplicity, completeness, and clarity [54] also covered various aspects of effective and practical design.

## Discussion

### Summary of Evidence

#### Overview

This scoping review synthesizes the existing evidence on the application of AI in health education for hypertension. A total of 24 studies met the inclusion criteria, and this scoping review yielded 3 key findings.

First, NLP and ML form the technological foundation of AI in hypertension health education. The study identified 3 application scenarios: rule-based health education, data-driven adaptive health education, and generative AI–driven health education. The evolutionary trajectory from rule-based to generative AI methods reflects the overall trend in the application of AI to hypertension health education, with research based on LLM surging since 2023. However, the use of generative AI in hypertension health education is still limited to the proof-of-concept phase. While some studies have used quantitative descriptive designs, none of them have undergone clinical validation through RCT.

Second, health education is generally embedded within multicomponent AI platforms rather than implemented as a standalone intervention; in all 6 RCTs, the educational module was provided concurrently with monitoring, reminder, or clinical decision support functions, making it impossible to assess the specific effects of the educational intervention in isolation.

Third, the included studies used a multidimensional evaluation framework covering the 4 domains of the Digital Health Scorecard, but significant asymmetry was observed. Technical metrics (accuracy, completeness, and readability) were reported most frequently, clinical outcome metrics (blood pressure and medication adherence) were reported in more than half of the studies, usability metrics (satisfaction and engagement) were reported less than the clinical outcome metrics, and cost assessments were rarely reported. This evaluation gradient, ranging from robust technical validation to a complete lack of economic analysis, indicates that the current evidence base is insufficient to support real-world implementation decisions. This is due to the absence of large-scale RCTs, short follow-up periods (mostly ≤12 weeks), and a general lack of cost-effectiveness data. These 3 points collectively constitute the 3 most urgent gaps in the current evidence base that need to be addressed.

#### Trends in the Application of AI in Hypertension Health Education

Rule-based health education systems, exemplified by early platforms such as the coaching app [34] and the digital therapeutic system [36], represent how AI technology was initially applied to deliver health education. Their strengths lie in content certainty and clinical interpretability: every piece of educational information can be traced back to predefined rules or validated knowledge structures, making these systems inherently traceable and suitable for integration into clinical workflows where transparency is paramount [37]. However, this certainty comes at the expense of flexibility; such systems cannot address novel queries outside their programmed knowledge domain and have limited capacity for personalization beyond predefined hierarchical criteria. This limitation echoes a longstanding critique in health education research that standardized, noncustomized materials often fail to meet the specific needs and circumstances of individual patients [59].

Data-driven adaptive approaches use ML to analyze user behavior and physiological data to enable dynamic educational functions. The personalized coaching platform developed by Leitner et al [45] is a prime example of this approach. By training personalized models, the platform identifies individual-specific associations between lifestyle factors and blood pressure. By providing dynamic feedback and adaptive health recommendations, these systems address the lack of personalization inherent in traditional rule-based frameworks. However, they also face their own challenges: achieving reliable personalization requires vast amounts of individual-level data, which raises concerns about data privacy. Furthermore, the effectiveness of these systems depends largely on users’ consistent use of monitoring devices, which may limit their applicability in settings where digital literacy or access to devices is limited [50].

The most transformative development has been the emergence of generative AI and LLM. Recent literature reviews on chronic disease management indicate that the use of LLMs in patient education has experienced explosive growth [22,23], a trend consistent with our findings: studies based on LLM have increased rapidly since 2023. By supporting multiturn natural language dialogue, these models overcome the one-way limitations of traditional health education, thereby enabling patients to actively seek information rather than passively receive it. However, LLMs also pose significant challenges to the reliability of educational content. As noted in evaluations of medical AI, LLMs remain prone to generating medical claims that appear plausible but lack evidence [60,61]. Within this review, LLM chatbot evaluations for hypertension education have demonstrated generally acceptable factual accuracy, yet revealed persistent concerns regarding readability and expert assessment consistency [42-44]. Patients, however, lack sufficient discernment, a factor that is particularly critical for the implementation of hypertension health education, which requires precise guidance on medication use and lifestyle adjustments [62,63]. To address this, recent studies advocate for the use of hybrid architectures to improve this situation by anchoring LLM to structured medical knowledge bases [64-66]. This aligns with the findings of this review, as multiple studies have used this approach to reduce hallucinations and enhance the accuracy of educational content [49,55,56]. Emerging evidence also suggests that performance gains can be achieved through prompt optimization alone. Li et al [67] demonstrated that structured prompt engineering strategies, particularly guidance-based and self-consistency approaches, substantially improved the accuracy and guideline adherence of LLM in hypertension treatment decision-making. This finding further highlights the potential of combining model architecture, knowledge integration, and prompt design to enhance the reliability and safety of AI-generated hypertension education.

#### The Outcome Measures Used to Evaluate the Effectiveness of AI Are Heterogeneous

This review found that classifying outcome metrics reveals heterogeneity in current evaluation practices. The technical dimension has consistently received significant attention, with its accuracy, completeness, readability, and reliability typically validated based on clinical guidelines or expert judgment [40-44,46]. This focus aligns with digital health validation pathways, which regard technical reliability as a prerequisite for clinical deployment [58]. However, a significant portion of the literature on LLM technologies for health education in this review remains at the proof-of-concept stage, assessing whether models can generate accurate information, rather than whether the information provided can change patient behavior or improve health outcomes. Currently, clinical validation of the use of LLM for hypertension health education is still in its early stages.

Blood pressure control is the most frequently studied clinical end point; however, most studies have relatively short follow-up periods, and only a few have used randomized designs to validate hard clinical end points based on established hypertension criteria [36,37,47]. Furthermore, the AI technologies used in these studies rarely involve LLM.

Even more striking is the complete absence of cost assessments across all 24 included studies. This omission aligns with evidence from implementation science, which indicates that even technically effective and user-friendly interventions struggle to achieve scale without data on implementation costs, technology lifecycle investments, and long-term health economic impacts [68,69]. Although usability evaluations covered user satisfaction, engagement rates, and system usability scales [38,50,54], these assessments primarily reflect short-term acceptance rather than long-term behavioral maintenance. Furthermore, research has found that content generated by LLM far exceeds the recommended reading level for patient education materials [41,43], raising fundamental equity concerns: if populations with lower health literacy, who are often older, less educated, and from lower socioeconomic backgrounds [70], cannot access AI-powered health education, these tools may exacerbate rather than alleviate existing inequalities in hypertension control.

#### Health Education as an Embedded Intervention Component

The AI systems included in this study share a notable characteristic that warrants special attention: health education rarely exists as a standalone intervention. Instead, it is embedded within multifunctional AI-driven platforms as one of their components [36,47,54]. This embedded nature has significant implications for effect attribution. When a multidimensional AI intervention significantly reduces blood pressure, it is impossible to distinguish the specific contribution of the educational component from the effects of the monitoring, reminder, or decision-support components. This challenge reflects a long-standing methodological debate regarding the evaluation of complex interventions, namely, that synergistic effects between components may exceed the sum of their individual contributions [71].

#### Multicultural Adaptation

This review found that the geographic distribution of the included studies exhibited distinct characteristics, with a significant concentration of research in China and the United States. This may reflect the policy momentum and technological investment in these 2 countries regarding the use of digital tools to manage hypertension. However, this concentration also raises an important question: whether the study findings are generalizable across different health care systems, reimbursement models, and sociocultural contexts. In total, 5 studies were conducted in low- and middle-income countries, 2 of which used relatively simple AI technologies rather than LLM. The study from Nigeria [50] demonstrated that a WhatsApp-based generative AI chatbot not only achieved high user satisfaction but also improved medication adherence in a resource-limited cardiology clinic. This study provides a key proof of concept but also highlights the significant gap in relevant evidence in low- and middle-income countries.

Research on multicultural and multilingual adaptability is almost entirely lacking. Only 2 studies have examined language-related performance differences, with one finding that blinded hypertension specialists rated English responses more favorably than Japanese responses [40], and this performance difference may align with the fact that current LLMs are primarily trained on English-based data. Beyond linguistic factors, although self-management behaviors for hypertension, including dietary choices, physical activity patterns, medication attitudes, and health care–seeking behaviors, are deeply influenced by cultural context [72,73], studies are needed to explore deeper dimensions of cultural adaptation.

#### Privacy and Security Considerations

This review found that only a few studies have systematically explored this topic. Montagna et al [52] proposed and compared various privacy-preserving architectures, revealing the inherent trade-offs between performance and data protection. Building on this work, Aguzzi et al [49] extended research on on-device deployment using enhanced RAG mini-language models, pointing the way toward the development of privacy-preserving AI tools that ensure data security without sacrificing performance. The design of digital health interventions must involve the active participation of the target population and be user-centered [74,75]. However, our findings indicate that this requirement has not yet been systematically implemented in AI-driven hypertension health education.

### Limitations

We must acknowledge that this scoping review has several limitations. First, we did not conduct a formal quality assessment or risk-of-bias assessment of the included studies. Although this approach is methodologically appropriate for scoping reviews aimed at mapping the evidence landscape rather than synthesizing effect sizes, it means that the strength of evidence from individual studies cannot be quantified. Therefore, our findings should be interpreted with caution. Second, our inclusion criteria were limited to peer-reviewed original research papers for which full-text access was available, thereby excluding preprints, conference abstracts, theses, and gray literature. This exclusion is particularly consequential for AI research, where technological advances are often first described in preprint repositories such as arXiv and medRxiv months or years before formal journal publication. Given the extremely short iteration cycles of AI technology, with major model updates occurring on the order of weeks to months, this limitation may systematically exclude the latest exploratory studies, negative results, and technical evaluations, introducing a temporal lag bias that could underestimate the current scope of AI applications in hypertension health education. Third, although our search strategy did not impose language restrictions, all 24 included studies were published in English-language journals, which may have systematically excluded relevant studies from non-English–speaking countries. Fourth, the included studies exhibited high clinical and methodological heterogeneity in terms of AI technology types, application scenarios, and outcome measures, making direct cross-study comparisons and quantitative meta-analyses impossible. Fifth, although categorizing application scenarios into 3 groups is conceptually useful, certain systems may possess features that span multiple categories. Sixth, the follow-up periods in most studies were relatively short, with only a few exceeding 12 weeks; therefore, evidence regarding the long-term maintenance of healthy behaviors, the sustainability of blood pressure control, and the reduction in cardiovascular events remains limited [76].

### Conclusions

This study used a scoping review methodology to examine the application of AI technologies in health education for hypertension. Most current reviews focus on broader topics, such as chronic disease management and the application of LLMs in hypertension care. Research findings indicate that AI technology plays a significant role in delivering health education on hypertension. In clinical practice, AI should be used as a tool to enhance, rather than replace, the health education provided by clinicians. The nature of educational components embedded within multifunctional AI platforms means that clinicians and developers must design systems in which educational features are purpose-built and assessable, rather than incidental. For the research community, the immediate priority is to bridge the gap between generative AI innovation and rigorous clinical validation. Future trials should prioritize hybrid architectures, combining the conversational flexibility of LLM with the reliability of structured medical knowledge bases via a RAG framework and adopt core outcome sets that encompass technical accuracy, behavioral change, and cardiovascular end points. For policymakers and health system planners, the widespread lack of cost-effectiveness data poses a fundamental obstacle to resource allocation decisions. Without evidence regarding implementation costs, technology lifecycle investments, and long-term health economic impacts, even technically superior interventions cannot be responsibly scaled up. Equity must be central to future efforts. Furthermore, research has primarily focused on high-income countries, with a near-total lack of studies on multicultural and multilingual adaptability. Combined with evidence suggesting that content generated by LLM exceeds the reading level recommended for patient education, these factors collectively increase the risk that AI-driven hypertension health education may exacerbate rather than narrow existing disparities in hypertension control. To address these interrelated challenges, standardized evaluation frameworks should be developed, cost-effectiveness benchmarks established, accessibility ensured for populations with varying levels of health literacy, and interventions validated across diverse sociocultural and resource settings, all to bring about substantial improvements in self-management for the hundreds of millions of people with hypertension worldwide.

## Supplementary material

10.2196/95596 Multimedia Appendix 1 Search strategy.

10.2196/95596 Multimedia Appendix 2 Characteristics of included studies.

10.2196/95596 Checklist 1 PRISMA-ScR checklist.

## Abbreviations

AI: artificial intelligence
LLM: large language model
ML: machine learning
MMAT: Mixed Methods Appraisal Tool
NLP: natural language processing
PRISMA: Preferred Reporting Items for Systematic Reviews and Meta-Analyses
PRISMA-ScR: Preferred Reporting Items for Systematic Reviews and Meta-Analyses Extension for Scoping Reviews
RAG: retrieval-augmented generation
RCT: randomized controlled trial
