# Supplementary material for: Application of AI in Hypertension Health Education: Scoping Review
Source: J Med Internet Res. 2026 Jul 15;28:e95596. doi: 10.2196/95596 (PMC13372264; doi:10.2196/95596)
Supplement: Multimedia Appendix 1 [file jmir-v28-e95596-s001.docx]

1. **Pubmed**

| **Search** | **Query** |
| --- | --- |
| #1 | "Hypertension"[Mesh] OR "Blood Pressure"[Mesh] OR hypertens*[tiab] OR "high blood pressure"[tiab] OR "elevated blood pressure"[tiab] OR "raised blood pressure"[tiab] OR "uncontrolled blood pressure"[tiab] OR "blood pressure control"[tiab] OR "blood pressure management"[tiab] OR "BP control"[tiab] OR "BP management"[tiab] OR "essential hypertension"[tiab] OR "primary hypertension"[tiab] OR "uncontrolled hypertension"[tiab] OR "resistant hypertension"[tiab] OR "arterial hypertension"[tiab] OR "hypertensive patient*"[tiab] OR "hypertensive individual*"[tiab] OR "hypertensive adult*"[tiab] OR "systolic hypertension"[tiab] OR "diastolic hypertension"[tiab] OR antihypertens*[tiab] OR "lowering blood pressure"[tiab] OR "high BP"[tiab] |
| #2 | "Artificial Intelligence"[Mesh] OR "Machine Learning"[Mesh] OR "Deep Learning"[Mesh] OR "Natural Language Processing"[Mesh] OR "Neural Networks, Computer"[Mesh] OR "Decision Support Systems, Clinical"[Mesh] OR "Expert Systems"[Mesh] OR "artificial intelligence"[tiab] OR AI[tiab] OR "machine learning"[tiab] OR "deep learning"[tiab] OR "neural network*"[tiab] OR "natural language processing"[tiab] OR NLP[tiab] OR "large language model*"[tiab] OR LLM[tiab] OR LLMs[tiab] OR "LLM-based"[tiab] OR "LLM-driven"[tiab] OR ChatGPT[tiab] OR GPT[tiab] OR "generative AI"[tiab] OR "generative artificial intelligence"[tiab] OR "generative pretrained transformer*"[tiab] OR "expert system*"[tiab] OR "knowledge graph*"[tiab] OR "knowledge base*"[tiab] OR "clinical decision support*"[tiab] OR "decision support system*"[tiab] OR "chatbot*"[tiab] OR "chat-bot*"[tiab] OR "conversational agent*"[tiab] OR "virtual assistant*"[tiab] OR "intelligent system*"[tiab] OR "recommender system*"[tiab] OR "predictive model*"[tiab] OR "prediction model*"[tiab] OR "random forest*"[tiab] OR "support vector machine*"[tiab] OR SVM[tiab] OR "reinforcement learning"[tiab] OR "transformer model*"[tiab] OR BERT[tiab] OR "bidirectional encoder"[tiab] OR "retrieval-augmented generation"[tiab] OR RAG[tiab] OR "text mining"[tiab] OR "speech recognition"[tiab] OR "fuzzy logic"[tiab] OR "Bayesian network*"[tiab] OR "ontology"[tiab] OR "supervised learning"[tiab] OR "unsupervised learning"[tiab] OR "data mining"[tiab] OR "pattern recognition"[tiab] OR "computational intelligence"[tiab] |
| #3 | "Health Education"[Mesh] OR "Patient Education as Topic"[Mesh] OR "Self Care"[Mesh] OR "Self-Management"[Mesh] OR "Patient Compliance"[Mesh] OR "Health Promotion"[Mesh] OR "Health Communication"[Mesh] OR "Health Literacy"[Mesh] OR "health education"[tiab] OR "patient education"[tiab] OR "health promotion"[tiab] OR "health communication"[tiab] OR "health information"[tiab] OR "patient information"[tiab] OR "patient teaching"[tiab] OR "patient counselling"[tiab] OR "patient counseling"[tiab] OR "self-management"[tiab] OR "self-management education"[tiab] OR "self care"[tiab] OR "self-care"[tiab] OR "lifestyle modification*"[tiab] OR "lifestyle intervention*"[tiab] OR "lifestyle change*"[tiab] OR "behavioral intervention*"[tiab] OR "behavioural intervention*"[tiab] OR "behavior change"[tiab] OR "behaviour change"[tiab] OR "health coaching"[tiab] OR "medication adherence"[tiab] OR "treatment adherence"[tiab] OR "therapeutic adherence"[tiab] OR "patient adherence"[tiab] OR "patient empowerment"[tiab] OR "health knowledge"[tiab] OR "patient knowledge"[tiab] OR "patient engagement"[tiab] OR "educational intervention*"[tiab] OR "educational program*"[tiab] OR "educational material*"[tiab] OR "health behavior"[tiab] OR "health behaviour"[tiab] OR "dietary advice"[tiab] OR "dietary education"[tiab] OR "exercise counseling"[tiab] OR "exercise education"[tiab] OR "lifestyle guidance"[tiab] OR "lifestyle advice"[tiab] OR "health advice"[tiab] OR "consumer health information"[tiab] |
| #4 | #1 AND #2 AND #3 |
| #5 | #4 Filters:from 2015/01/01–2026/06/05 |

1. **Embase**

| **Search** | **Query** |
| --- | --- |
| #1 | 'hypertension'/exp OR 'blood pressure'/exp OR 'hypertens*':ti,ab,kw OR 'high blood pressure':ti,ab,kw OR 'elevated blood pressure':ti,ab,kw OR 'raised blood pressure':ti,ab,kw OR 'uncontrolled blood pressure':ti,ab,kw OR 'blood pressure control':ti,ab,kw OR 'blood pressure management':ti,ab,kw OR 'bp control':ti,ab,kw OR 'bp management':ti,ab,kw OR 'essential hypertension':ti,ab,kw OR 'primary hypertension':ti,ab,kw OR 'uncontrolled hypertension':ti,ab,kw OR 'resistant hypertension':ti,ab,kw OR 'arterial hypertension':ti,ab,kw OR 'hypertensive patient*':ti,ab,kw OR 'hypertensive individual*':ti,ab,kw OR 'hypertensive adult*':ti,ab,kw OR 'systolic hypertension':ti,ab,kw OR 'diastolic hypertension':ti,ab,kw OR 'antihypertens*':ti,ab,kw OR 'lowering blood pressure':ti,ab,kw OR 'high bp':ti,ab,kw |
| #2 | 'artificial intelligence'/exp OR 'machine learning'/exp OR 'deep learning'/exp OR 'natural language processing'/exp OR 'artificial neural network'/exp OR 'clinical decision support system'/exp OR 'expert system'/exp OR 'artificial intelligence':ti,ab,kw OR 'ai':ti,ab,kw OR 'machine learning':ti,ab,kw OR 'deep learning':ti,ab,kw OR 'neural network*':ti,ab,kw OR 'natural language processing':ti,ab,kw OR 'nlp':ti,ab,kw OR 'large language model*':ti,ab,kw OR 'llm':ti,ab,kw OR 'llms':ti,ab,kw OR 'llm-based':ti,ab,kw OR 'llm-driven':ti,ab,kw OR 'chatgpt':ti,ab,kw OR 'gpt':ti,ab,kw OR 'generative ai':ti,ab,kw OR 'generative artificial intelligence':ti,ab,kw OR 'generative pretrained transformer*':ti,ab,kw OR 'expert system*':ti,ab,kw OR 'knowledge graph*':ti,ab,kw OR 'knowledge base*':ti,ab,kw OR 'clinical decision support*':ti,ab,kw OR 'decision support system*':ti,ab,kw OR 'chatbot*':ti,ab,kw OR 'chat-bot*':ti,ab,kw OR 'conversational agent*':ti,ab,kw OR 'virtual assistant*':ti,ab,kw OR 'intelligent system*':ti,ab,kw OR 'recommender system*':ti,ab,kw OR 'predictive model*':ti,ab,kw OR 'prediction model*':ti,ab,kw OR 'random forest*':ti,ab,kw OR 'support vector machine*':ti,ab,kw OR 'svm':ti,ab,kw OR 'reinforcement learning':ti,ab,kw OR 'transformer model*':ti,ab,kw OR 'bert':ti,ab,kw OR 'bidirectional encoder':ti,ab,kw OR 'retrieval-augmented generation':ti,ab,kw OR 'rag':ti,ab,kw OR 'text mining':ti,ab,kw OR 'speech recognition':ti,ab,kw OR 'fuzzy logic':ti,ab,kw OR 'bayesian network*':ti,ab,kw OR 'ontology':ti,ab,kw OR 'supervised learning':ti,ab,kw OR 'unsupervised learning':ti,ab,kw OR 'data mining':ti,ab,kw OR 'pattern recognition':ti,ab,kw OR 'computational intelligence':ti,ab,kw |
| #3 | 'health education'/exp OR 'patient education'/exp OR 'self care'/exp OR 'self care'/exp OR 'patient compliance'/exp OR 'health promotion'/exp OR 'medical information'/exp OR 'health literacy'/exp OR 'health education':ti,ab,kw OR 'patient education':ti,ab,kw OR 'health promotion':ti,ab,kw OR 'health communication':ti,ab,kw OR 'health information':ti,ab,kw OR 'patient information':ti,ab,kw OR 'patient teaching':ti,ab,kw OR 'patient counselling':ti,ab,kw OR 'patient counseling':ti,ab,kw OR 'self-management':ti,ab,kw OR 'self-management education':ti,ab,kw OR 'self care':ti,ab,kw OR 'self-care':ti,ab,kw OR 'lifestyle modification*':ti,ab,kw OR 'lifestyle intervention*':ti,ab,kw OR 'lifestyle change*':ti,ab,kw OR 'behavioral intervention*':ti,ab,kw OR 'behavioural intervention*':ti,ab,kw OR 'behavior change':ti,ab,kw OR 'behaviour change':ti,ab,kw OR 'health coaching':ti,ab,kw OR 'medication adherence':ti,ab,kw OR 'treatment adherence':ti,ab,kw OR 'therapeutic adherence':ti,ab,kw OR 'patient adherence':ti,ab,kw OR 'patient empowerment':ti,ab,kw OR 'health knowledge':ti,ab,kw OR 'patient knowledge':ti,ab,kw OR 'patient engagement':ti,ab,kw OR 'educational intervention*':ti,ab,kw OR 'educational program*':ti,ab,kw OR 'educational material*':ti,ab,kw OR 'health behavior':ti,ab,kw OR 'health behaviour':ti,ab,kw OR 'dietary advice':ti,ab,kw OR 'dietary education':ti,ab,kw OR 'exercise counseling':ti,ab,kw OR 'exercise education':ti,ab,kw OR 'lifestyle guidance':ti,ab,kw OR 'lifestyle advice':ti,ab,kw OR 'health advice':ti,ab,kw OR 'consumer health information':ti,ab,kw OR 'patient-facing':ti,ab,kw |
| #4 | #1 AND #2 AND # 3  Refine by Publication Years：2015/01/01–2026/06/05 |

1. **Web Of Science**

| **Search** | **Query** |
| --- | --- |
| #1 | TS=(hypertens* OR "high blood pressure" OR "elevated blood pressure" OR "raised blood pressure" OR "uncontrolled blood pressure" OR "blood pressure control" OR "blood pressure management" OR "BP control" OR "BP management" OR "essential hypertension" OR "primary hypertension" OR "uncontrolled hypertension" OR "resistant hypertension" OR "arterial hypertension" OR "hypertensive patient*" OR "hypertensive individual*" OR "hypertensive adult*" OR "systolic hypertension" OR "diastolic hypertension" OR antihypertens* OR "lowering blood pressure" OR "high BP") |
| #2 | TS=("artificial intelligence" OR AI OR "machine learning" OR "deep learning" OR "neural network*" OR "natural language processing" OR NLP OR "large language model*" OR LLM OR LLMs OR "LLM-based" OR "LLM-driven" OR ChatGPT OR GPT OR "generative AI" OR "generative artificial intelligence" OR "generative pretrained transformer*" OR "expert system*" OR "knowledge graph*" OR "knowledge base*" OR "clinical decision support*" OR "decision support system*" OR chatbot* OR "chat-bot*" OR "conversational agent*" OR "virtual assistant*" OR "intelligent system*" OR "recommender system*" OR "predictive model*" OR "prediction model*" OR "random forest*" OR "support vector machine*" OR SVM OR "reinforcement learning" OR "transformer model*" OR BERT OR "bidirectional encoder" OR "retrieval-augmented generation" OR RAG OR "text mining" OR "speech recognition" OR "fuzzy logic" OR "Bayesian network*" OR ontology OR "supervised learning" OR "unsupervised learning" OR "data mining" OR "pattern recognition" OR "computational intelligence") |
| #3 | TS=("health education" OR "patient education" OR "health promotion" OR "health communication" OR "health information" OR "patient information" OR "patient teaching" OR "patient counselling" OR "patient counseling" OR "self-management" OR "self-management education" OR "self care" OR "self-care" OR "lifestyle modification*" OR "lifestyle intervention*" OR "lifestyle change*" OR "behavioral intervention*" OR "behavioural intervention*" OR "behavior change" OR "behaviour change" OR "health coaching" OR "medication adherence" OR "treatment adherence" OR "therapeutic adherence" OR "patient adherence" OR "patient empowerment" OR "health knowledge" OR "patient knowledge" OR "patient engagement" OR "educational intervention*" OR "educational program*" OR "educational material*" OR "health behavior" OR "health behaviour" OR "dietary advice" OR "dietary education" OR "exercise counseling" OR "exercise education" OR "lifestyle guidance" OR "lifestyle advice" OR "health advice" OR "consumer health information" OR "patient-facing") |
| #4 | #1 AND #2 AND # 3 |
| #5 | Refine by Publication Years：2015/01/01–2026/06/05 |

1. **Cochrane Library**

| **Search** | **Query** |
| --- | --- |
| #1 | MeSH descriptor: [Hypertension] explode all trees |
| #2 | ((hypertens* OR "high blood pressure" OR "elevated blood pressure")):ti,ab,kw |
| #3 | #1 OR #2 |
| #4 | MeSH descriptor: [Artificial Intelligence] explode all trees |
| #5 | MeSH descriptor: [Machine Learning] explode all trees |
| #6 | MeSH descriptor: [Natural Language Processing] explode all trees |
| #7 | MeSH descriptor: [Neural Networks, Computer] explode all trees |
| #8 | MeSH descriptor: [Knowledge Bases] explode all trees |
| #9 | MeSH descriptor: [Expert Systems] explode all trees |
| #10 | (("artificial intelligence" OR "machine learning" OR "deep learning" OR "natural language processing" OR "neural network" OR "knowledge base" OR "knowledge bases" OR "expert system" OR "expert systems" OR "generative artificial intelligence" OR "large language model" OR "large language models" OR "LLM" OR "LLMs" OR chatbot OR chatbots OR "conversational AI" OR "ChatGPT" OR "generative AI" OR "knowledge graph" OR "knowledge graphs" OR "retrieval augmented generation" OR "RAG")):ti,ab,kw |
| #11 | #4 OR #5 OR #6 OR # 7 OR #8 OR #9 OR #10 |
| #12 | MeSH descriptor: [Health Education] explode all trees |
| #13 | MeSH descriptor: [Patient Education as Topic] explode all trees |
| #14 | MeSH descriptor: [Self Care] explode all trees |
| #15 | (("health education" OR "patient education" OR "health promotion" OR "self-management" OR "health literacy" OR "lifestyle guidance" OR "medication adherence" OR "blood pressure monitoring" OR "behavioral intervention" OR "behavioral interventions" OR "behavioural intervention" OR "behavioural interventions")):ti,ab,kw |
| #16 | #12 OR #13 OR #14 OR #15 |
| #17 | #3 AND #11 AND #16 |
| #18 | Date:2015-2026 |

1. **CINHAL**

| **Search** | **Query** |
| --- | --- |
| #1 | (MH "Hypertension+") |
| #2 | XB (hypertens* OR "high blood pressure" OR "elevated blood pressure") |
| #3 | #1 OR #2 |
| #4 | (MH "Artificial Intelligence+") |
| #5 | (MH "Machine Learning+") |
| #6 | (MH "Natural Language Processing") |
| #7 | (MH "Neural Networks (Computer)") |
| #8 | (MH "Expert Systems") |
| #9 | XB ("artificial intelligence" OR "machine learning" OR "deep learning" OR "natural language processing" OR "neural network" OR "knowledge base" OR "knowledge bases" OR "expert system" OR "expert systems" OR "generative artificial intelligence" OR "large language model" OR "large language models" OR "LLM" OR "LLMs" OR chatbot* OR "conversational AI" OR "ChatGPT" OR "generative AI" OR "knowledge graph" OR "knowledge graphs" OR "retrieval augmented generation" OR "RAG") |
| #10 | #4 OR #5 OR #6 OR #7 OR #8 OR #9 |
| #11 | (MH "Health Education+") |
| #12 | (MH "Patient Education+") |
| #13 | (MH "Self Care+") |
| #14 | XB ("health education" OR "patient education" OR "health promotion" OR "self-management" OR "health literacy" OR "lifestyle guidance" OR "medication adherence" OR "blood pressure monitoring" OR "behavioral intervention" OR "behavioral interventions" OR "behavioural intervention" OR "behavioural interventions") |
| #15 | #11 OR #12 OR #13 OR #14 |
| #16 | #3 AND #10 AND #15 |
| #17 | Date Range:01/2015-05/2026 |

1. **Scopus**

| **Search** | **Query** |
| --- | --- |
| #1 | TITLE-ABS-KEY(hypertens* OR "high blood pressure" OR "elevated blood pressure") |
| #2 | TITLE-ABS-KEY("artificial intelligence" OR "machine learning" OR "deep learning" OR "natural language processing" OR "neural network" OR "knowledge base" OR "knowledge bases" OR "expert system" OR "expert systems" OR "generative artificial intelligence" OR "large language model" OR "large language models" OR "LLM" OR "LLMs" OR chatbot* OR "conversational AI" OR "ChatGPT" OR "generative AI" OR "knowledge graph" OR "knowledge graphs" OR "retrieval augmented generation" OR "RAG") |
| #3 | TITLE-ABS-KEY("health education" OR "patient education" OR "health promotion" OR "self-management" OR "self care" OR "self-care" OR "health literacy" OR "lifestyle guidance" OR "medication adherence" OR "blood pressure monitoring" OR "behavioral intervention*" OR "behavioural intervention*") |
| #4 | #1 AND #2 AND #3 |
| #5 | Date:2015-2026 |
